# A comprehensive analysis of malaria transmission in Brazil

**DOI:** 10.1080/20477724.2019.1581463

**Published:** 2019-03-04

**Authors:** Bianca C. Carlos, Luisa D. P. Rona, George K. Christophides, Jayme A. Souza-Neto

**Affiliations:** a School of Agricultural Sciences, Department of Bioprocesses and Biotechnology, Central Multiuser Laboratory, São Paulo State University (UNESP), Botucatu, Brazil; b Institute of Biotechnology, São Paulo State University (UNESP), Botucatu, Brazil; c Department of Life Sciences, Imperial College London, London, UK; d Department of Cell Biology, Embryology and Genetics, Federal University of Santa Catarina (UFSC), Florianópolis, Brazil; e National Council for Scientific and Technological Development (INCT-EM, CNPq), National Institute of Science and Technology in Molecular Entomology, Rio de Janeiro, Brazil

**Keywords:** Malaria transmission, Amazon rainforest, Atlantic rainforest, *Plasmodium vivax*, *Plasmodium falciparum*, *Anopheles*, Malaria hotspots, bromeliad malaria

## Abstract

Malaria remains a serious public health problem in Brazil despite a significant drop in the number of cases in the past decade. We conduct a comprehensive analysis of malaria transmission in Brazil to highlight the epidemiologically most relevant components that could help tackle the disease. We consider factors impacting on the malaria burden and transmission dynamics including the geographical occurrence of both autochthonous and imported infections, the distribution and abundance of malaria vectors and records of natural mosquito infections with *Plasmodium*. Our analysis identifies three discrete malaria transmission systems related to the Amazon rainforest, Atlantic rainforest and Brazilian coast, respectively. The Amazonian system accounts for 99% of all malaria cases in the country. It is largely due to autochthonous *P. vivax* and *P. falciparum* transmission by mosquitoes of the Nyssorhynchus subgenus, primarily *Anopheles darlingi*. Whilst *P. vivax* transmission is widespread, *P. falciparum* transmission is restricted to hotspot areas mostly in the States of Amazonas and Acre. This system is the major source of *P. vivax* exportation to the extra-Amazonian regions that are also affected by importation of *P. falciparum* from Africa. The Atlantic system comprises autochthonous *P. vivax* transmission typically by the bromeliad-associated mosquitoes *An. cruzii* and *An. bellator* of the *Kerteszia* subgenus. *An. cruzii* also transmits simian malaria parasites to humans. The third, widespread but geographically fragmented, system is found along the Brazilian coast and comprises *P. vivax* transmission mainly by *An. aquasalis*. We conclude that these geographically and biologically distinct malaria transmission systems require specific strategies for effective disease control.

## Malaria situation in Brazil: past and present

Between 2010 and 2015, there has been a significant worldwide drop in malaria clinical cases and mortality, reaching 21% and 31% in Africa and 29% and 37% in South America, respectively. Nonetheless, according to the World Health Organization (WHO), there were 212 million estimated cases and 429,000 deaths in 2016, 90% of them in Africa, and 71% of them concerning children under the age of five [1]. The disease remains a major driver of poverty, with malaria-associated treatment and disability-adjusted life years (DALYs) accounting for over 12 billion US dollars per year in Africa alone [2].

Brazil has a centenary history of fighting malaria. In 1905, the first antimalarial campaign was carried out in Brazil, during the construction of the port of Santos [3–5]. Despite the lack of detailed records, it is estimated that as many as 10,000 people died from malaria during the construction of the Madeira-Mamore Railroad in the State of Rondônia in 1907–1912, where immunologically naive immigrants worked [4].

The introduction of *An. arabiensis* in Northeastern Brazil by boats arriving from Africa in the 1930s is held responsible for some 14,000 deaths in 1938–1939. At the time, *An. arabiensis* was not recognized as a separate species; and all the species comprising the, what is now known, *An. gambiae sensu lato* species complex were collectively called *An. gambiae* [6]. With about 20% of the population being infected, a national campaign assisted by the Rockefeller Foundation began in 1938, and the African mosquito was eliminated in less than two years [3,7–10]. Nonetheless, malaria remained a major national issue with more than six million infections per year in the 1940s. Therefore, a new control campaign established the National Malaria Service and employed DDT (dichloro-diphenyl-trichloroethane) and chloroquine for both mosquito and parasite control, respectively [7,11]. This effectively led to a consistent reduction in the total number of malaria cases in Brazil and almost total elimination of the disease in the extra-Amazonian region in the 1950s-60s [8,11].

**Figure 1.**
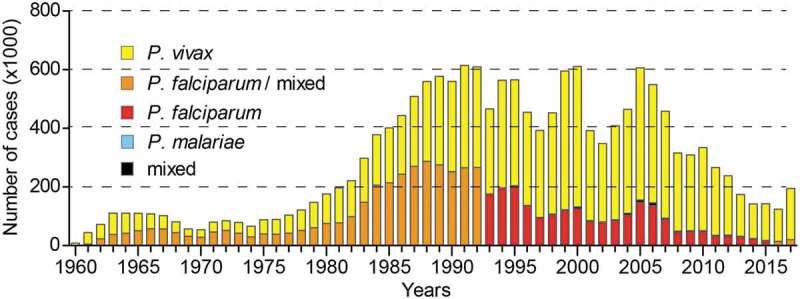
Number of malaria cases in Brazil between 1960 and 2017. The *Plasmodium* species responsible are shown with different colors. Until 1992, *P. falciparum* mixed infections are pooled with P. falciparum mono-infections. Source of data: Pan American Health Organization (PAHO), http://www.paho.org.

After this period of decrease in the number of cases, new malaria outbreaks were recorded in the 1960s-70s (Figure 1). During this time, the Amazonian region was heavily colonized with over one million people from all over Brazil moving to the area. Their activities included development of agriculture, cattle farming, mining, energy farming, and road infrastructure [5,7,12], which were associated with significant interferences with the Amazonian ecosystem including deforestation and changes in river flows, lakes and marshes [13]. This greatly favored the expansion of vector populations and resulted in a progressive increase in malaria cases [5]. Consequently, the entire Northern Region experienced a dramatic increase in malaria cases from 52,000 in 1970 to over 600,000 in the early 1990s. In response, the National Program for Malaria Control was launched, focusing on early diagnosis and treatment through an increased number of local health care facilities. At the time, infections with *P. falciparum* and *P. vivax* displayed similar rates, and the program soon led to reduction of *P. falciparum* cases, even though the total number of cases remained relatively high [5,7,11,14,15].

The last 30 years saw fluctuations in the number of malaria cases, with peaks of around 600,000 cases in 1998–2000 and 2004–2006 due to reasons that are not fully understood but thought to be linked to environmental changes, poor management of fish farming, human migration and *P. vivax* recrudescence [7,8]. This situation prompted a renewed, intensified effort for disease control and prevention that led to a reduction from approximately 600,000 cases in 2005 to 143,000 cases in 2014 (Figure 1). Between 2014 and 2016, malaria remained relatively stable, but the significant rise in 2017 to 194,000 cases is particularly alarming and highlights the fragility of the malaria control program. This dramatic jump may be connected with a shift in priorities toward control of other vector-borne diseases including dengue, Zika and chikungunya [11,16–18]. Moreover, the impact of environmental phenomena that influence the Amazon rainforest climate such as the 2014–2016 El Niño-Southern Oscillation that promoted an extended period of extreme drought [19] should also be considered. In fact, one may argue that the relatively low number of cases in 2014–2016 is due to El Niño, and that the increase of cases in 2017 denotes the actual impact of the control programs.

**Figure 2.**
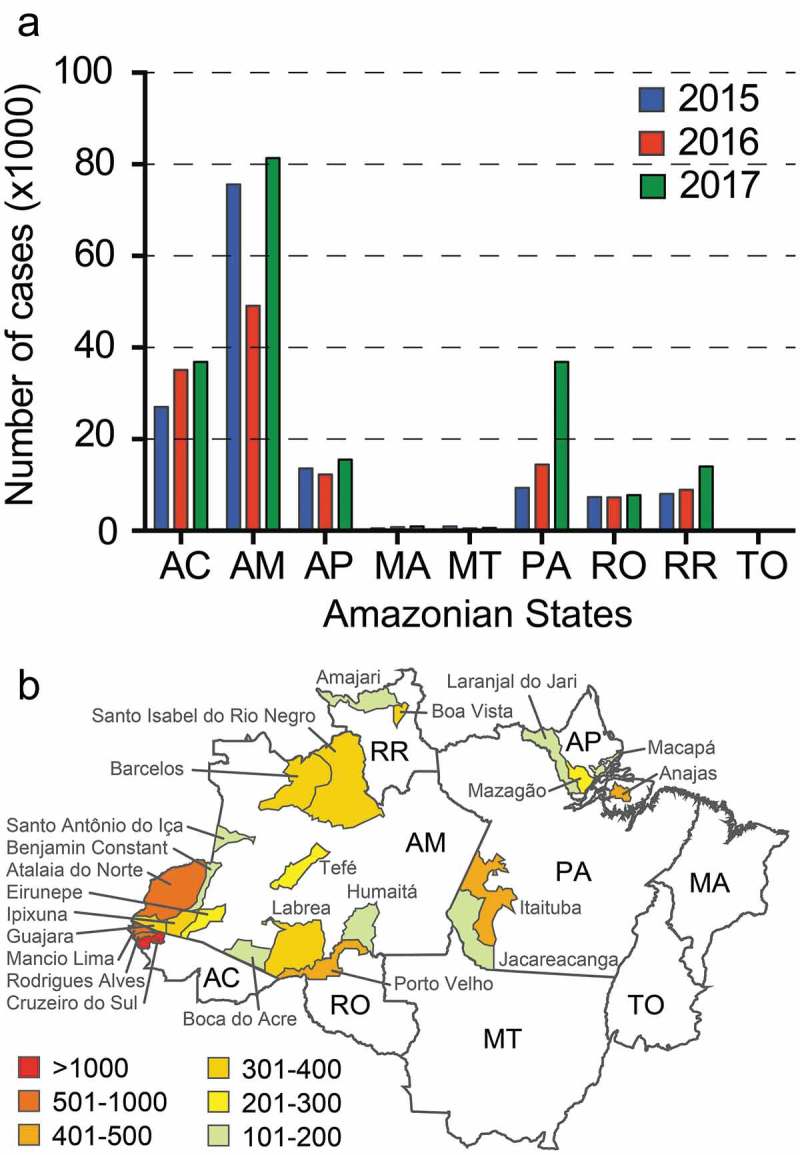
(a) Number of malaria cases in the Brazilian Amazonian States in 2015 (blue bars) and 2016 (red bars) and 2017 (green bars). (b) *P. falciparum* cases in municipalities of the Amazonian States. The mean number of cases in 2015 and 2016 is depicted with colour coding explained in the key. Municipalities with over 100 average cases are shown. AC, Acre; AM, Amazonas; AP, Amapá; MA, Maranhão; MT, Mato Grosso; PA, Pará; RO, Rondônia; RR, Roraima; TO, Tocantins.

Malaria is a serious public health problem in the Amazonian region where 99% of all cases are recorded (Figure 2(a)). The high malaria incidence and burden impact significantly on the economic growth and development of the affected Amazonian populations, namely in the States of Acre (AC), Amazonas (AM), Amapá (AP), Pará (PA), Rondônia (RO) and Roraima (RR). The remaining three States of the Amazonian region, i.e. Maranhão (MA), Mato Grosso (MT) and Tocantins (TO) together account for only about 1% of the total number of cases [20].

## *P. falciparum* hotspots and the *P. vivax* burden

In 2015–2016, the States of Amazonas and Acre together reported 60–70% of malaria cases in the Amazonian Region and the entire country. In particular, *P. falciparum* cases in these two States were as high as 75% of all *P. falciparum* cases in the country. Of these, about 60% were in the neighboring municipalities of Cruzeiro do Sul (AC), Rodrigues Alves (AC), Mâncio Lima (AC), Atalaia do Norte (AM), Guajara (AM), Ipixuna (AM), Eirunepe (AM) and Benjamin Constant (AM) (Figure 2(b)). Five of these municipalities bordering Peru inflicted the majority of the burden. Interestingly, these and other municipalities in the region, including Labrea (AM), Tarauaca (AC), Boca do Acre (AC) and Santo Antônio do Iça (AM) experienced a dramatic rise in *P. falciparum* cases in the past 6 years with a peak in 2013–2014. That year, *P. falciparum* cases in Cruzeiro do Sul reached 6,286, a number about twice as high as in 2009 (2,796) and 2016 (2,370). Today, Cruzeiro do Sul alone accounts for about 30% of all *P. falciparum* cases in the country and together with its neighboring Rodrigues Alves and Mâncio Lima account for about 50% of all *P. falciparum* cases. Other *P. falciparum* hotspots are Porto Velho (RO), Itaituba (PA) and Anajas (PA) [21].

The reasons for the persistently high and relatively increasing rates of *P. falciparum* cases in the westernmost part of the Brazilian Amazon are poorly understood and thought to be multiple. The high disease transmission dynamics are thought to be affected by the extensive anthropogenic environmental changes in conjunction with the booming of fish farming in the region, which are likely to have extended the habitats and increased the density of vectors, rendering vector control tools insufficient. Human migration between municipalities, states and countries may also be an important factor and is analyzed later in this paper.

Even though *P. falciparum* is responsible for the majority of severe malaria cases and deaths, the greatest DALYs burden is inflicted by *P. vivax* that accounts for about 90% of all the cases in Brazil [22,23]. Controlling *P. vivax* infections presents many challenges, owing to the *P. vivax* unique biological characteristics that include early blood stream production of gametocytes that are infectious to mosquitoes, low parasitaemia that can be undetectable by light microscopy, hypnozoites that can become active after months and sometimes years of latency, and asymptomatic infections that are common favoring the maintenance of the parasite reservoir. A recent study in Remansinho (Labrea, AM) revealed that 65% of the cases tested positive for *P. vivax* with PCR presented no malaria symptoms, while 54% of them could not be diagnosed by conventional microscopy [24]. Strikingly, nearly all of the PCR-positive individuals carried infective gametocytes. A direct correlation between *P. vivax* parasitaemia and the presence of symptoms was observed. Considering that only microscopy-confirmed cases are treated, both asymptomatic and oligosymptomatic (few and moderate clinical symptoms) cases are a major challenge that needs urgent attention.

Asymptomatic and oligosymptomatic cases are also a problem in States spanning the Atlantic rainforest, where atypical autochthonous cases, mostly diagnosed as *P. vivax*, occur every year. These cases often show low parasitaemia of very short duration that cure spontaneously [25–29]. In the State of Espirito Santo, high percentages of *IgM* and *IgG* antibodies for *P. vivax, P. malariae* and *P. falciparum* were detected among people with no obvious malaria symptoms, suggesting elevated exposure rates [30]. Similar data were obtained in the State of São Paulo [27–29]. Additionally, the presence of *P. falciparum* and *P. vivax* was verified in healthy blood donors from the São Paulo State, which were associated with forest environment and fragmentation [31].

## Malaria importation and migration

Human population movement is a key challenge in tackling malaria transmission in Brazil. Whilst temporary movement, mostly associated with business, holidays or social visitations, could contribute to small-scale outbreaks in malaria-free areas, long-term and continuous migration can significantly contribute to the observed and stable malaria transmission dynamics. We propose that the latter is mostly associated with *P. vivax* malaria, and to a lesser extent to *P. falciparum* that is restricted to short distance movement (e.g. between neighboring municipalities or States), and is related to poverty that forces families to move in search of work and better opportunities [3].

**Figure 3.**
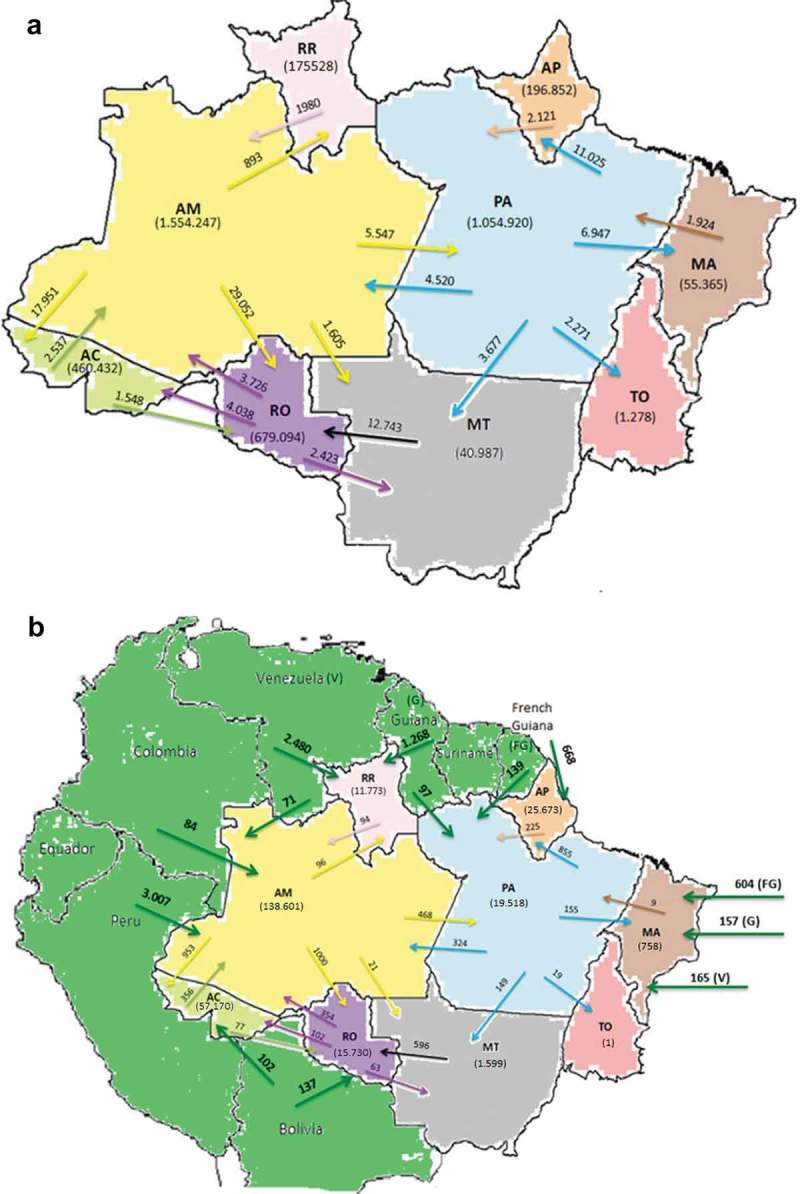
Number of imported malaria cases in Brazilian Amazonian States. (a) Total number of imported cases between Amazonian States in the period of 2003 to February 2016. (b) Total number of imported cases from neighboring countries in the period of 2014–2015. The start of each arrow indicates the likely origin of infection and the arrowhead indicates the number of locally diagnosed imported cases. AC, Acre; AM, Amazonas; AP, Amapá; MA, Maranhão; MT, Mato Grosso; PA, Pará; RO, Rondônia; RR, Roraima; TO, Tocantins; FG, French Guiana; G, Guiana; V, Venezuela. The total number of autochthonous cases is each State is shown in brackets. The data were taken from SIVEP, Epidemiological surveillance information system – 2016.

Although the majority of malaria cases in the Brazilian Amazon are autochthonous, case importation between States can provide important insights into disease transmission dynamics (Figure 3(a)). Between 2003 and February 2016, the State of Amazonas, which presented most autochthonous malaria cases in the country, exported the highest number of cases to neighboring States, mainly Rondônia and Acre. Thus, human migration from the State of Amazonas to the semi-urban settings of Acre, presumably combined with poor living standards of migrants, may be partly responsible for the increase of *P. falciparum* cases in Acre seen in the last decade. This pattern of malaria migration was followed by the States of Pará and Mato Grosso, the latter exporting about one fifth of all its cases, mostly to the State of Rondônia.

Importation of malaria from neighboring countries may also play an important role in malaria transmission in the Brazilian Amazon (Figure 3(b)). For example, in the State of Maranhão, the number of cases imported from the French Guiana, Guiana and Venezuela in 2014–15 was equal to the number of autochthonous cases and those imported from the State of Pará together. At the same time, the number of cases imported to the State of Roraima, primarily from Venezuela and secondarily from Guiana, was one fourth of all the cases. Overall, the number of cases imported from Peru, primarily to the State of Amazonas, has been the highest compared to those from other South American countries in recent years (3,007 in 2014–2015). We presume that the most affected municipalities are those directly bordering Peru, which are the same as those presented above as a *P. falciparum* hotspot (see Figure 2(b)). This may also be connected with the *P. falciparum* hotspot in the State of Acre that, as commented upon previously, receives a large number of immigrants from the State of Amazonas. This analysis may highlight a route of malaria migration in the region.

**Figure 4.**
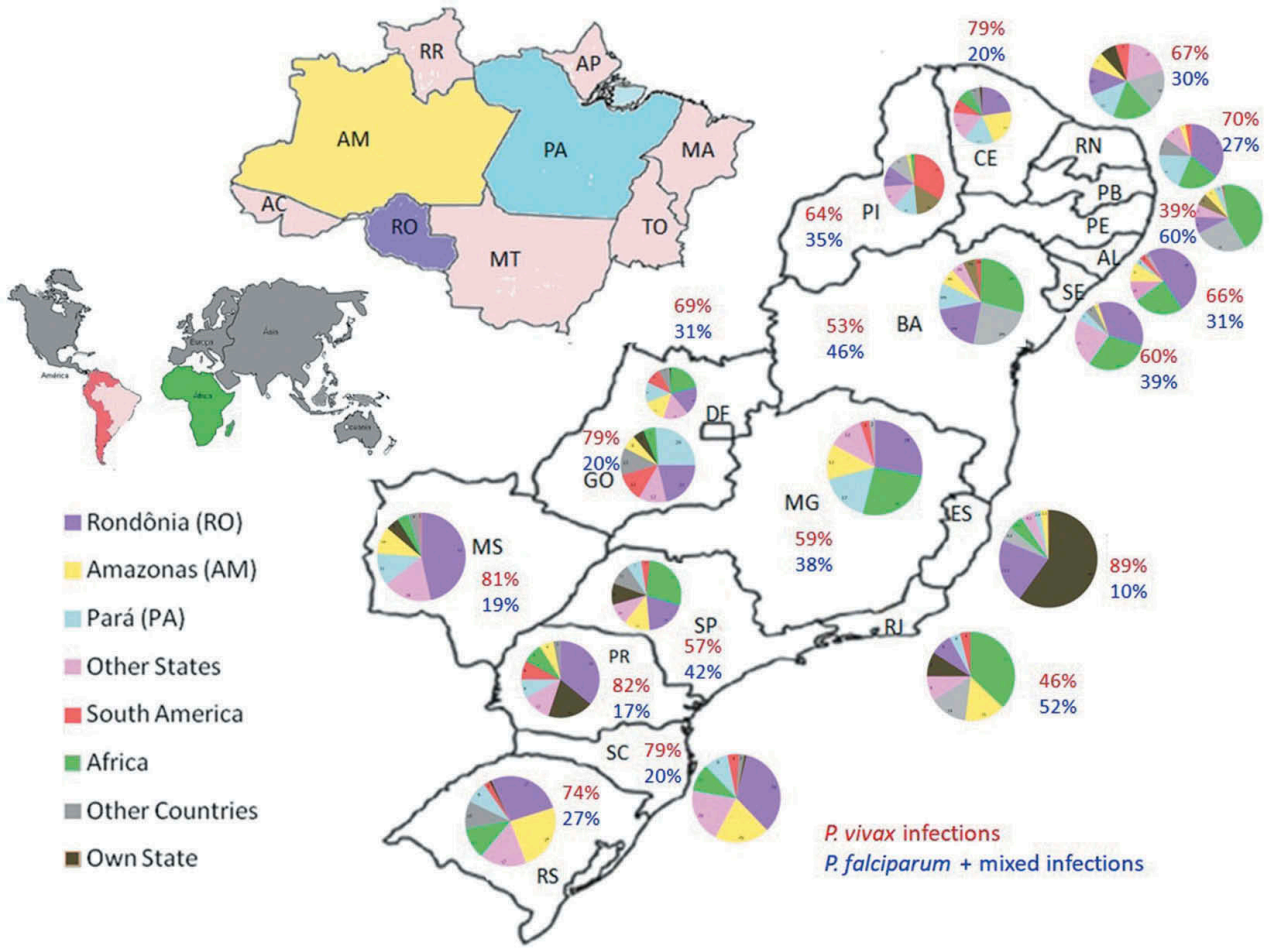
Map of probable origin of imported cases in extra-Amazonian Region. Colors represents the different origins and the numbers show the proportion of infections by *P. vivax* (red color) and *P. falciparum* plus mixed infections (blue color). The graphs were made considering the total cases between 2007 to February 2016 (SINAN -Notification of injury information system – 2016) http://dtr2004.saude.gov.br/sinanweb/).

Imported malaria cases can also have serious implications in regions that are free of malaria or record a low number of autochthonous cases, as is the case of the extra-Amazonian Region. The human population of the extra-Amazonian Region is about 6 times larger than the Amazonian population, estimated at about 178 million people [32]. As discussed in subsequent sections, competent malaria vectors are found in all the States in this region, especially in coastal areas as well as areas encompassing the Atlantic rainforest. With the exception of the State of Espírito Santo, which recorded many autochthonous cases in the period of 2007–2016, imported cases in the remaining extra-Amazonian States ranged from 81% to 100% of the total number of diagnosed cases (Figure 4 and Table 1). Although the percentages vary between States and indeed regions, most of these cases originated from the Amazonian States of Rondônia, Amazonas and, to a lesser extent, Pará. Specifically, imported cases from Rondônia appeared to be evenly distributed across the extra-Amazonian region with States such as Pernambuco, Sergipe, Santa Catarina and Mato Grosso do Sul registering more than 30% of all their cases as originating from Rondônia, while imported cases from Amazonas were mostly recorded in Southeastern and Southern States.

**Table 1.** Malaria cases in states of the extra-Amazonian region in the period 2007–2016.

	Northeast								Midwest			Southeast				South		
Possible origin of infection and parasite species	AL	BA	CE	PB	PE	PI	SE	RN	DF	GO	MS	ES	MG	RJ	SP	PR	RS	SC
Rondônia (%)	48	19	22.6	36	8	11	35	12	18	21	47	21.2	28	8	19	36	27	34
Pará (%)	3	10	17.2	19	4	13	5	13	13	26	12	2.4	17	4	7	8	8	9
Amazonas (%)	9	6	20.9	3	5	2	2	7	14	6	10	2.3	12	15	12	6	24	20
Other States (%)	10	5	16.2	9	6	12	23	19	16	12	18	4.3	12	9	10	12	17	20
South America (%)	3	2	8.1	3	1	33	0	6	10	12	1	0.3	3	4	4	8	2	4
Africa (%)	24	29	7.7	20.3	44	2	30	17	21	5	4	4.6	26	37	28	8	11	10
Other Countries (%)	2	24	5.7	9.4	26	11	5	18	7	12	4	4.9	2	14	10	3	10	2
Autocthonous/Introduced (%)	0	5	1.7	0	6	15	0	7	1	5	5	60	0	9	10	19	1	1
Total Malaria Cases	58	252	297	64	225	608	43	113	286	707	227	655	859	890	1830	836	157	269
Total Imported Cases	58	239	292	64	212	514	43	105	284	670	216	262	857	807	1652	680	155	266
Total Imported Cases (%)	100	95	98	100	94	85	100	93	99	95	95	40	100	91	90	81	99	99
Total Malaria Cases	58	252	297	64	225	608	43	113	286	707	227	655	859	890	1830	836	157	269
*P. vivax* (%)	66	53	79	70	39	64	60	67	69	79	81	89	59	46	57	82	74	79
*P. falciparum* (%)	29	42	16	22	59	27	30	25	30	15	14	8	27	50	34	14	24	16
*P. vivax/P. falciparum*(%)	2	4	4	5	1	8	9	5	1	5	5	2	11	2	8	3	3	4
*P. ovale* (%)	0	0	1	0	0	0	0	1	0	0	0	1	3	1	0.3	0	0	0
*P. malariae* (%)	3	0	0	3	0	0	0	2	0	0	0	1	0	1	0.8	0	0	0

PI: Piauí; CE: Ceara, RN: Rio Grande do Norte, PB: Paraíba, PE: Pernambuco, AL: Alagoas, SE: Sergipe, BA: Bahia, GO: Goiás, DF: Distrito Federal, MG, Minas Gerais, ES: Espírito Santo, RJ: Rio de Janeiro, SP: São Paulo, MS, Mato Grosso do Sul, PR: Paraná, SC: Santa Catarina, RS: Rio Grande do Sul.

Importantly, imported cases from Africa, mainly from Angola, accounted for the majority of internationally imported cases in the extra-Amazonian regions (Figure 4). Whereas *P. vivax* was by far responsible for most of the imported cases, *P. falciparum* was the main imported parasite in states where Africa represented the major source of imported cases, including the States of Pernambuco, Rio de Janeiro, Sergipe, Bahia, São Paulo, Minas Gerais and Paraíba. With the decrease in the number of *P. falciparum* infections imported from other regions, the relative impact of African-imported cases in the total number of *P. falciparum* diagnosed cases in the extra-Amazonian region has become more pronounced in recent years. Specially, the States of São Paulo, Pernambuco, Bahia, Paraíba and Sergipe reported 55–67% *P. falciparum* imported cases in 2014–2015. Malaria importation from Africa presents a great challenge for the reintroduction of chloroquine-resistant parasites. In Brazil, chloroquine is still used in endemic areas for treating *P. vivax* infections, even though resistance has already been documented in both *P. falciparum* and *P. vivax* [33–35].

## Malaria vectors in Brazil

Known malaria vector mosquitoes belong to the genus *Anopheles*, subgenera *Anopheles, Cellia, Kerteszia* and *Nyssorhynchus*. Of these, *Cellia*, the most popular subgenus, is only found in the Old World and includes some of the most notorious vectors such as *An. gambiae, An. coluzzi, An. arabiensis, An. funestus*, and *An. stephensi*. In Brazil, 61 species belonging to the other three subgenera were identified, largely using morphological criteria [36]. It is now understood that some of these species are indeed species complexes [37–41].

**Figure 5.**
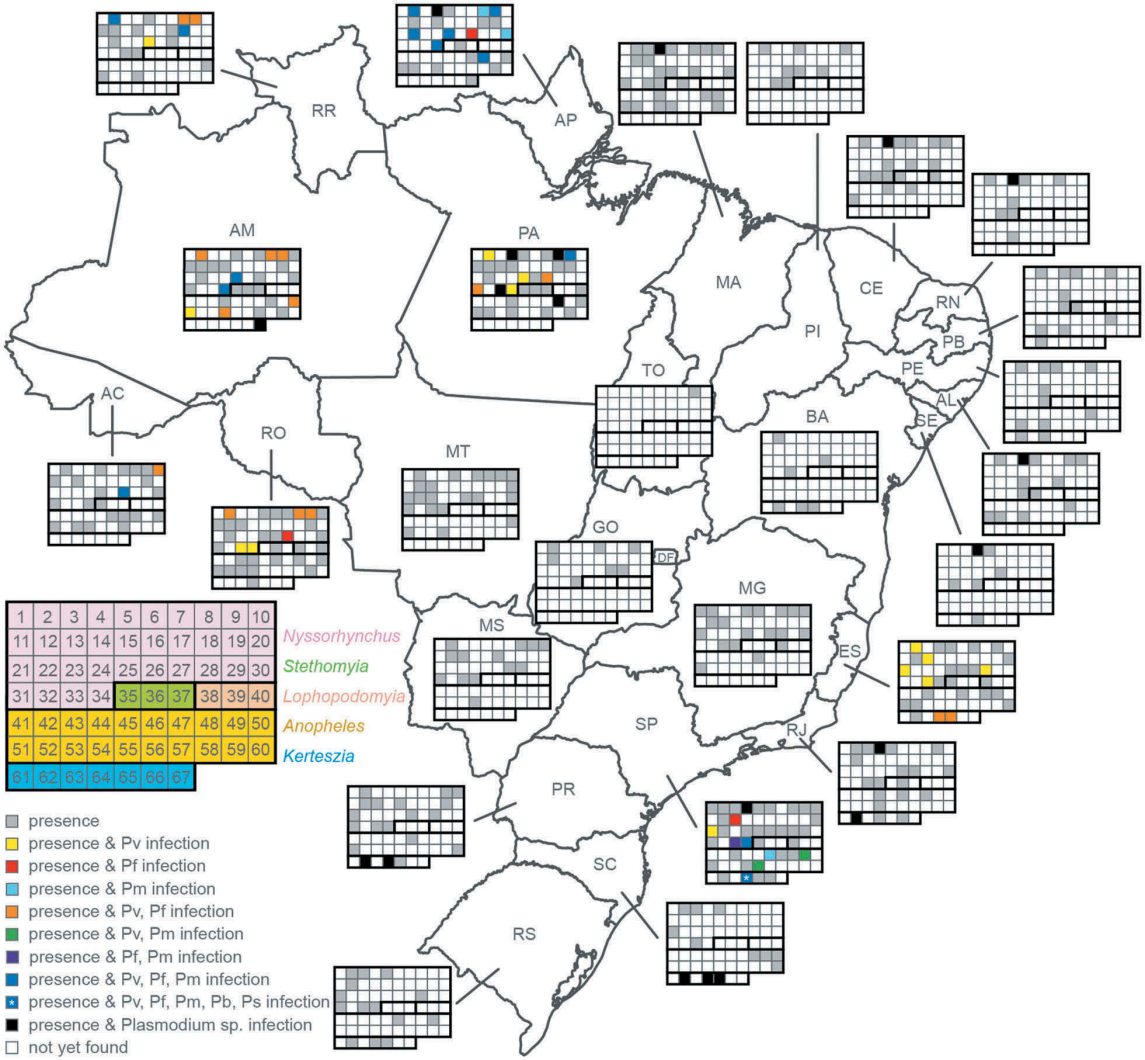
Distribution and vectorial capacity of *Anopheles* mosquitoes. States are abbreviated as follows: AC, Acre; AM, Amazonas; AP, Amapá; MA, Maranhão; MT, Mato Grosso; PA, Pará; RO, Rondônia; RR, Roraima; TO, Tocantins; PI, Piauí; CE, Ceará; RN, Rio Grande do Norte; PB, Paraíba; PE, Pernambuco; AL, Alagoas; SE, Sergipe; BA, Bahia; MG, Minas Gerais. SP, São Paulo; ES, Espírito Santo; RJ, Rio de Janeiro; GO, Goiás; DF, Distrito Federal; MS, Mato Grosso do Sul; PR, Paraná; SC, Santa Catarina; RS, Rio Grande do Sul. Shaded (including colored) squares depict recorded presence of a mosquito species, while the color indicates detection of one or more parasite species as shown in the key. 1, *An. albertoi*; 2, *An. albitarsis* s.l.; 3, *An. antunesi*; 4, *An. aquasalis*; 5, *An. argyritarsis*; 6, *An. arthuri*; 7, *An. benarrochi*; 8, *An. braziliensis*; 9, *An. darlingi*; 10, *An. deaneorum*; 11, *An. dunhami*; 12, *An. evansae*; 13, *An. galvaoi*; 14, *An. goeldii*; 15, *An. guarani*; 16, *An. halophylus*; 17, *An. ininii*; 18, *An. janconnae*; 19, *An. konderi*; 20, *An. lanei*; 21, *An. lutzii*; 22, *An. marajoara*; 23, *An. nigritarsis*; 24, *An. noroestensis*; 25, *An. nunesztovari* s.l.; 26, *An. oryzalimnetes*; 27, *An. oswaldoi* s.l.; 28, *An. parvus*; 29, *An. pristinus*; 30, *An. rangeli*; 31, *An. rondoni*; 32, *An. sawyer*; 33, *An. strode*; 34, *An. triannulatus* s.l.; 35, *An. kompi*; 36, *An. nimbus*; 37, *An. thomasi*; 38, *An. gilesi*; 39, *An. pseudotibiamaculatus*; 40, *An. squamifemur*; 41, *An. anchietai*; 42, *An. bustamentei*; 43, *An. costai*; 44, *An. eiseni*; 45, *An. evandroi*; 46, *An. fluminensis*; 47, *An. forattinii*; 48, *An. intermedius*; 49, *An. maculipes*; 50, *An. mattogrossensis*; 51, *An. mediopunctatus*; 52, *An. minor*; 53, *An. neomaculipalpus*; 54, *An. peryassui*; 55, *An. pseudomaculipes*; 56, *An. pseudipunctipennis*; 57, *An. punctimacula*; 58, *An. ranchoui*; 59, *An. shannoni*; 60, *An. tibiamaculatus*; 61, *An. bambusicolus*; 62, *An. bellator*; 63, *An. boliviensis*; 64, *An. cruzii*; 65, *An. homunchulus*; 66, *An. laneanus*; 67, *An. neivai*. Pv, *P. vivax*; Pf, *P. falciparum*, Pm, *P. malariae*, Pb, *P. braziliensis*; Ps, *P. simium.*

We carried out a comprehensive literature review of the distribution of species or species complexes and their capacity to transmit malaria. The results are schematically summarized in Figure 5 and presented in full in Supplementary Data S1. In addition to the aforementioned four subgenera, the distribution of six species belonging to the subgenera *Stethomyia* and *Lophopodomyia* is also presented for completeness. We recognize that this is not an exhaustive record and that the absence of a species from any certain State compared to another may only be due to under-sampling; for example, in the State of Tocantins only *An. darlingi* is reported to be present to date. The same is likely true for the detection of *Plasmodium* infection in a vector species. In addition, we believe there are some inaccuracies in vector species identification such as the detection of *An. cruzii* in the Amazonian State of Rondônia. Finally, in some cases the exact location of a certain species could not be verified due to lack of information and/or inaccessibility to old literature. In such cases, we report the species in Figure 5 as generally present in Brazil without specifying the state in which it was found.

The highest vector diversity is documented in the Amazonian and the Southeastern regions for both of the *Nyssorhynchus* and the *Anopheles* subgenera. Of the former subgenus, *An. darlingi* is reported in all the Amazonian States, and *An. albitarsis, An. braziliensis, An. argyritarsis, An. nunesztovari, An. oswaldoi* and *An. triannulatus* are also found in all the Amazonian States but Tocantins (most likely due to limited sampling). The same species are present in Southeastern States that are run by the Atlantic rainforest, and some are also found further south. Most of these species persist but are more scarcely detected in the Midwestern and the non-Amazonian Northeastern States, while other species of the *Nyssorhynchus* subgenus become more prevalent. Of the *Anopheles* subgenus, *An. mattogrossensis, An. mediopunctatus* and *An. peryassui* are also found in all the Amazonian States but Tocantins, with no or scarce detection in the remaining Northeastern and the Midwestern States. Some of the species persist in Southeastern and Southern States, again suggestive of their forest-related habitats. Species of the *Kerteszia* subgenus, especially *An. bellator* and *An. cruzii*, are widely distributed in Southeastern and Southern States but scarcely found in Amazonian States.

Twenty-seven (27) out of 37 of these mosquito species were tested positive to date for natural *Plasmodium* infection using ELISA detection of the circumsporozoite protein (CSP), microscopy and/or PCR. Of these, 16 were of the *Nyssorhynchus* subgenus, 7 of the *Anopheles* subgenus and 4 of the *Kerteszia* subgenus. The majority of mosquitoes for which the species of *Plasmodium* harbored was determined were found positive for more than one of the three human parasites circulating in Brazil, i.e. *P. vivax, P. falciparum* and *P. malariae* (Figure 5 and Supplementary Data S1). Of these, *An. albitarsis, An. braziliensis, An. darlingi, An. janconnae, An. marajoara, An. nunesztovari, An. oswaldoi, An. strodei* and *An. triannulatus* of the *Nyssorhynchus* subgenus and *An. intermedius* of the *Anopheles* subgenus were found positive for all three parasites, while, in the State of São Paulo, *An. cruzii* of the *Kerteszia* subgenus was additionally found positive for the simian parasites, *P. simium* and *P. brazilianum*.

With regards to the geographical distribution of malaria-positive mosquitoes, 10 species were found in the States of Amapá and Pará, 9 in Amazonas and São Paulo, 7 in Espírito Santo, 6 in Rondônia, 5 in Roraima, 3 in Santa Catarina, 2 in Acre, 2 in Rio de Janeiro, 2 in Paraná, and 1 in each of Alagoas, Ceará, Maranhão, Rio Grande do Norte and Sergipe. To the best of our knowledge, no data is available for the remaining States, including the Amazonian States of Mato Grosso, where numerous autochthonous cases are recorded every year, and Tocantins, while the data for Acre and Maranhão are outdated. Characteristically, there is no report of infected *An. darlingi* in the State of Acre where it is thought to be the primary vector, altogether reinforcing our view that malaria transmission in the Brazilian Amazon is poorly studied and understood.

Very little is also known about insecticide resistance among vector species. We were able to identify only a recent report for the State of Amapá demonstrating that *An. darlingi* is susceptible to cypermethrin, deltamethrin, and alpha-cypermethrin, whereas *An. marajoara* displayed higher tolerance to these chemicals [42].

**Figure 6.**
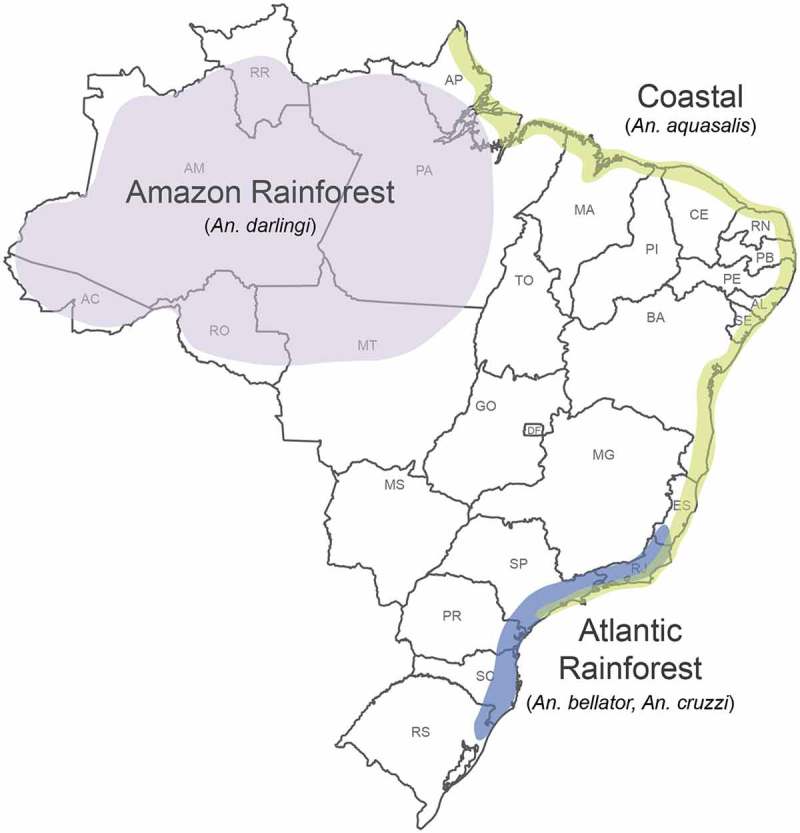
Map of malaria transmission systems in Brazil. The three geographically and biologically distinct transmission systems are maintained by different mosquito vectors and have different eco-epidemiological characteristics. The Amazon rainforest transmission system is the most important (99% of cases) and primarily involves *An. darlingi*. The Atlantic rainforest transmission system involves the bromeliad-associated mosquitoes *An. bellator* and *An. cruzii*. The ‘coastal’ transmission system mostly involves *An. aquasalis.*

In conclusion, when analyzed together with the malaria case incidents, these data reveal three discrete malaria transmission systems in Brazil (Figure 6). The first is related to the Amazonian rainforest, which is largely supported by species of the *Nyssorhynchus* subgenus, while species of the *Anopheles* subgenus play a lesser role, mostly confined to the State of Amazonas. The second, geographically more limited and epidemiologically of lesser significance system coincides with the Atlantic rainforest, where species of the *Kerteszia* subgenus appear to play an important but not the sole role. The third, less discernible, not continuous and mostly *P. vivax* transmission system can be identified as overlapping with the Brazilian coast, especially in the Northeastern and Southeastern territories. It is vectored by *An. aquasalis* of the *Nyssorhynchus* subgenus, which breeds exclusively in brackish waters [43–46]. This mosquito is mostly zoophilic and found to attack humans only when it reaches high densities [36]. Below we analyze in more detail the two main transmission systems, i.e. the Amazonian and the Atlantic rainforests transmission systems.

## The Amazon rainforest transmission system

The primary vector in the Amazonian rainforest is *An. darlingi*, which is highly anthropophilic with crepuscular biting activity usually extending throughout the night [47–50]. It can be both endophagic and exophagic, and mostly rests inside houses after blood feeding, although the increased use of indoor insecticides may have selected *An. darlingi* populations with outdoor resting behavior [43]. Immature stages are usually found in clean and deep water surrounded by vegetation, hiding in the shade until adult emergence [36]. However, larvae have also been found in poorly maintained or abandoned fishponds, which is thought to contribute to urban malaria in some States such as Acre [51]. The detected rates of *An. darlingi* natural infection by *Plasmodium* mostly ranges from 0 to 10% but can reach over 20% depending on the area, year and season [52–57].

Across the Amazon, *An. darlingi* is often found in greater abundance and higher proportion than any other species [50,58]. In Amapá, the peak of *An. darlingi* abundance is seen between June and August, during the transition between the rainy and dry seasons [59]. However, in Rondônia, two genetically distinct *An. darlingi* groups were found to be associated with different peak periods: the first in the rainy season and the second in dry environment [60].

Besides *An. darlingi*, other *Nyssorhynchus* species also involved in the Amazon malaria transmission system are *An. albitarsis* s.l., *An. braziliensis, An. nuneztovari* s.l., *An. oswaldoi* s.l. and *An. triannulatus* s.l., which are mostly of local relevance. *An. marajoara* and *An. janconnae*, for example, which belong to the *An. albitarsis* s.l. complex [61], are considered important vectors in Amapá and Roraima, respectively, where they are found in great abundance and positive for *Plasmodium* [47,50,55,62,63]. *Anopheles braziliensis, An. nuneztovari* s.l, *An. oswaldoi* s.l. and *An. triannulatus* s.l. are present in most Amazonian States and considered as secondary vectors because they are mostly zoophilic and exophilic [36,38,47,49,50,54–57,64–71].

## The Atlantic rainforest transmission system

Most autochthonous cases in the extra-Amazonian region in the period of 2007–2016 were recorded in the State of Espírito Santo (ca. 400 cases), corresponding to 60% of the State’s total number of cases (Figure 5 and Table 1). A high number of autochthonous cases were also detected in other states along the southern Atlantic coast, including Rio de Janeiro (ca. 80 cases), São Paulo (ca.180 cases) and Paraná (ca. 160 cases), suggesting that autochthonous transmission in these areas is likely associated with the Atlantic rainforest.

As mentioned above, *An. aquasalis* is present all along the Brazilian coast and may account in part for the autochthonous cases mentioned above. According to Coutinho [72], an infection rate of up to 4% was recorded in *An. aquasalis* mosquitoes captured in the State of Rio de Janeiro. This mosquito is also held responsible for the last large outbreak in the State of São Paulo in 1986, which occurred in the urban coastal area of Guarujá and resulted in 24 autochthonous cases.

In recent years, the majority of cases in these areas were associated with the Atlantic rainforest, where mosquitoes of the *Kerteszia* subgenus are thought to be the main vectors. Bromeliad malaria is a term coined by Downs & Pittendrigh [73] to describe malaria transmitted by vectors breeding in water accumulating in bromeliad plants that are abundantly found across the Atlantic rainforest. Its transmission characteristics differs from that of Amazonian malaria, whose main vectors are water pool or paddle breeding mosquitoes. Bromeliad malaria, firstly detected during the construction of the São Paulo-Santos railway in 1898 [74–76], became endemic in the 1940s reaching some 40,000 cases annually in southern Brazil, mostly in the States of Paraná and Santa Catarina. Vector control measures including removal of bromeliads and deforestation followed by the introduction of eucalyptus trees were highly effective in reducing cases to less than 100 by 1982 [43]. Despite continuing vector control measures, transmission still occurs in or near the forest where competent bromeliad-breeding vectors, including *An. cruzii* and *An. bellator*, are found. This, combined with the high rates of imported malaria cases from Amazonian States and Africa, make such areas potential hotspots for seasonal outbreaks.

Besides being a vector of human malaria, *An. cruzii* is also a natural vector of the simian malaria parasites, *P. simium* and *P. brasilianum* [36,65,77–79]. Owing to its aggressive and promiscuous feeding behavior, *An. cruzii* is held responsible for transmission of simian malaria to humans in or near fragments of the Atlantic forest. For years, simian *Plasmodium* species were thought to routinely infect humans and lead to infections of low parasitaemia that cure spontaneously [77,79,80]. Extensive genetic, morphological and immunological similarities between *P. brasilianum* and *P. malariae*, and between *P. simium* and *P. vivax* are thought to have led to misdiagnosis between simian and human malaria. Indeed, using molecular markers, Brasil et al. [81] identified 28 malaria cases infected by *P. simium* in the State of Rio de Janeiro in 2015 and 2016, which were originally diagnosed as *P. vivax*.

## Concluding remarks

- There are three geographically and biologically distinct malaria transmission systems in Brazil, each maintained by different mosquito vectors and having different eco-epidemiological characteristics, thus requiring tailored control strategies.
- The first and most important system is related to the Amazon rainforest and primarily involves *An. darlingi*. The second, less prominent but stable system is associated with the Atlantic rainforest and involves the bromeliad-associated mosquitoes *An. cruzii* and *An. bellator*. The third, less discernible system, responsible for alarming *P. vivax* outbreaks, is found along the Brazilian coast and involves *An. aquasalis*.
- 99% of malaria cases in Brazil are recorded in the Amazon rainforest transmission system, affecting mostly the States of Amazonas and Acre. This system is poorly studied and understood.
- While *P. vivax* malaria remains the leading infection, the presence of *P. falciparum* malaria hotspots requires special attention. The most prominent hotspot involves the westernmost municipalities of Acre and Amazonas.
- Human migration is thought to have a major influence on malaria transmission dynamics throughout Brazil.
- High incidence of malaria case importation is detected between Amazonian states but also from neighboring South American countries, particularly *P. falciparum* cases from Peru. The latter may contribute to the *P. falciparum* hotspot referred to above.
- In the extra-Amazonian region, malaria is mostly due to *P. vivax* imported from the Brazilian Amazon whereas Africa is the main origin of cases in states where *P. falciparum* is the majorly imported parasite.
- Importation of malaria parasites from Africa may represent an epidemiologically relevant route for the reintroduction of chloroquine-resistance.
